# EPA Prevents the Development of Abdominal Aortic Aneurysms through Gpr-120/Ffar-4

**DOI:** 10.1371/journal.pone.0165132

**Published:** 2016-10-20

**Authors:** Ryo Kamata, Batmunkh Bumdelger, Hiroki Kokubo, Masayuki Fujii, Koichi Yoshimura, Takafumi Ishida, Mari Ishida, Masao Yoshizumi

**Affiliations:** 1 Department of Cardiovascular Physiology and Medicine, Graduate School of Biomedical and Health Sciences, Hiroshima University, Hiroshima, Japan; 2 Department of Surgery and Clinical Science, Graduate School of Medicine, Yamaguchi University, Ube, Japan; 3 Department of Cardiovascular Medicine, Fukushima Medical University, Fukushima, Japan; Yokohama City University Graduate School of Medicine, JAPAN

## Abstract

Abdominal aortic aneurysms (AAAs), which commonly occur among elderly individuals, are accompanied by a risk of rupture with a high mortality rate. Although eicosapentaenoic acid (EPA) has been reported to prevent AAA formation, the mechanism by which EPA works on vascular smooth muscle cells is unknown. This study aimed to investigate the mechanism by which orally-administered EPA prevents the formation of severe AAAs that develop in *Osteoprotegerin* (*Opg*) knockout (KO) mice. In the CaCl_2_-induced AAA model, EPA attenuated the enhanced progression of AAAs in *Opg-*KO mice, including the increase in aortic diameter with destruction of elastic fibers in the media. Immunohistochemical analyses showed that EPA reduced the phosphorylation of transforming growth factor beta-activated kinase-1/Map3k7 (Tak-1) and c-Jun NH2-terminal kinase (JNK), as well as the expression of *Matrix metalloproteinase-9* (*Mmp-9*) in the media of the aorta. In smooth muscle cell cultures, rh-TRAIL-induced activation of the Tak-1-JNK pathway and increase in *Mmp-9* expression were inhibited by EPA. Moreover, GW9508, a specific ligand for G-protein coupled receptor (Gpr)-120/Free fatty acid receptor (Ffar)-4, mimicked the effects of EPA. The effects of EPA were abrogated by knockdown of the *Gpr-120/Ffar-4* receptor gene. Our data demonstrate that the Trail-Tak-1-JNK-Mmp-9 pathway is responsible for the enhancement of AAAs in *Opg-*KO mice, and that EPA inhibits the Tak-1-JNK pathway by activating Gpr-120/Ffar-4, which results in the attenuation of AAA development.

## Introduction

An abdominal aortic aneurysm (AAA) is a condition in which the abdominal aorta is expanded, leading to rupture with a high mortality rate. Destruction of medial elastic fibers and infiltration of macrophages into AAA lesions are considered as the main causes of AAA formation[1]. Degradation of aortic medial elastic fibers may be induced by imbalances between the proteolytic activities of matrix metalloproteinases, such as MMP-2 and MMP-9, and their inhibitors, the tissue inhibitors of metalloproteinases (Timp-1). The expression of both is reported to be elevated during aneurysm development[2–6]. It has been reported that MMPs are induced by activation of the JNK pathway[5,7,8] in macrophages and vascular smooth muscle cells (SMCs), and that inhibition of the JNK pathway results in AAA regression[7]. Pro-inflammatory cytokines, such as tumor necrosis factor (TNF)-α and interleukin (IL)-1, are known to activate the JNK pathway[9].

OPG, which is secreted from osteoblasts, promotes bone formation by acting as a decoy receptor for the receptor activator of nuclear factor kappa-B ligand (Rankl), a factor that promotes the differentiation and activation of osteoclasts during osteogenesis[10,11]. OPG is expressed in vascular SMCs, and plays roles in preventing arterial calcification and stabilizing plaque formation in animal models[12–14]. We recently reported that Opg prevents the development of AAAs, given the reduced formation of AAAs with complete disruption of medial elastic fibers in *Opg*-KO mice[15]. TNF-related apoptosis inducing ligand (Trail), which was increased in *Opg*-KO mice, induced the expression of Mmp-9 through JNK, and of Timp-1 through nuclear factor (NF)-κB pathways, in aortic SMCs. Since OPG functions as a decoy receptor for Trail, we concluded that Opg prevents AAA formation through its antagonistic effect on Trail.

Eicosapentaenoic acid (EPA), an omega-3 polyunsaturated fatty acid (ω-3 PUFA), showed great promise in the prevention of cardiovascular diseases through its anti-inflammatory effects[16–18]. In particular, EPA attenuated the development of AAAs through suppression of tissue remodeling and inhibition of macrophage-mediated inflammation [19] [20]. The molecular mechanism underlying EPA’s anti-inflammatory effects in the vascular system is not yet fully understood. Recently, EPA has been reported to function as a ligand for Gpr-120/Ffar-4. Activation of Gpr-120/Ffar-4 was shown to induce the release of glucagon-like peptide (GLP)-1 into the intestine[21], and also exhibited a possible anti-inflammatory effect by inhibiting JNK and NF-κB pathways in macrophages and adipocytes[22]. It is unknown whether Gpr-120/Ffar-4 is expressed in vascular SMCs, and whether it plays a role in AAA formation.

Here, we report that orally-administered EPA prevents the enlargement of AAAs in *Opg-*KO mice in a CaCl_2_-induced AAA model. We found the size of the abdominal aorta to be significantly reduced in *Opg-*KO mice fed an EPA (+) diet. In vascular SMCs, EPA and GW9508, agonists of Gpr-120/Ffar-4, significantly reduced the TRAIL-induced expression of *Mmp-9* by inhibiting the JNK pathway. This suppression was reversed by knock-down of *Gpr-120/Ffar-4* expression. Our findings suggest that EPA can prevent the enlargement of AAAs by activating Gpr-120/Ffar-4-mediated signaling in aortic SMCs.

## Materials and Methods

### Generation of a Mouse Model for AAAs

The experiment protocol was approved by the Committee of Animal Experimentation at Hiroshima University (A08-32) and carried out in accordance with this protocol. All surgeries were performed under sodium phenobarbital anesthesia, and efforts were made to minimize suffering during and after surgery. Animals were sacrificed by anesthetic overdose with sodium phenobarbital. Wild-type and *Opg*-KO male mice of the C57BL/6J strain (CLEA Japan, Inc.) were used. AAA was induced by the CaCl_2_ method as described in our previous report[15]. Briefly, we added 50 μL of 0.5 M CaCl_2_ to a small piece of cotton and placed it on the aortic wall for 15 min. The CaCl_2_-treated area was limited to 1/2 to 1/3 of the circumference surface. Characteristic of our peri-aortic CaCl_2_ application is minimized invasion of the aorta. The F1-diet, which does not contain fish meat (Funabashi Farm Co., Ltd.), was mixed 5% (wt/wt) with EPA (provided by Mochida Pharmaceutical Co., Ltd.) and fed to mice starting two weeks before AAA induction up to the point of aorta examination.

### Morphological Examination

Paraffin-embedded aortic tissues were used to generate 6 μm-thick sections that were subsequently subjected to hematoxylin & eosin (H&E), Elastica van Gieson (EVG), Aniline Blue-Azan, or von Kossa staining using standard protocols. The maximum aortic diameter, including thickened adventitia with inflammation, was measured as the external aortic diameter. For widths of the medial layers of the aorta, the maximum values were measured on the sides to which CaCl_2_ was applied. The means of maximum and minimum measurements of the internal cross-sections of the aorta reflect the internal diameter of the aorta. All measurements were performed using Photoshop (Adobe Systems) and Image-J (National Institutes of Health; NIH).

### Immunohistochemistry

Prior to staining, sections were pre-incubated in antigen retrieval solution (pH 5.2) at 90°C for 45 minutes, based on the manufacturer’s instructions (Dako). For double-immunohistochemistry, antigen-retrieved sections were blocked with 1% bovine serum albumin in 0.1% Tween-phosphate buffered solution (PBS), incubated with a combination of primary antibodies, and subsequently incubated with appropriate secondary antibodies, including Alexa Fluor 488 or 555 dyes and conjugated anti-mouse, -rat, -rabbit, or -goat antibodies (donkey, 1:500; Life Technologies). Sections were then counterstained with 4'-6-diamidino-2-phenylindole (DAPI). Antibodies for Mmp-9 (goat polyclonal, 1:40; R&D Systems), Trail (rabbit polyclonal, 1:100; Abcam), phosphorylated-SAPK/JNK (rabbit, 1:100; Cell Signaling), α-smooth muscle actin (SMA; mouse, monoclonal, 1:100; Sigma Aldrich), and a polyclonal antibody to GPR-120/FFAR-4 (rabbit, 1:1000; Cell Signaling), were used as primary antibodies. Signals were detected using a DMI4000 fluorescence microscope (Leica Microsystems). Photoshop (Adobe Systems) or Image-J (NIH) software was used to calculate the signal area, which was automatically detected based on color, as well as the entire intima-medial area, selected based on morphology. The percent positive area for pJNK and Mmp-9 was calculated by dividing the area by the entire intima-medial region.

### Real-Time PCR

Total RNA was isolated using TRIzol (Invitrogen). Reverse transcription was performed with the ReverTra Ace^®^ qPCR RT Kit (TOYOBO). Real-time PCR was conducted using SYBR Premix Ex Taq II (Takara Bio Inc. and Kapa Biosystems Inc.). Intensities of PCR products were measured and analyzed using Opticon (MJ Research). The following primers were used for *Mmp-9*: forward 5’-GCCCTGGAACTCACACGACA-3’ and reverse 5’-TTGGAAACTCACACGCCAGAAG-3’. Amplification conditions were 5 s at 95°C, 20 s at 60°C, and 15 s at 72°C, for 49 cycles. Internal controls included *G3pdh* or *β-actin*. To detect messenger ribonucleic acid (mRNA) of *Gpr-120/Ffar-4*, RNA extracts were amplified by PCR using the following primers: forward 5’-TGTCCCAACAAGACTACCGAC-3’ and reverse 5’-ACAGTATGGGGTTTAGGGCAG-3’, with the following conditions: 30 s at 94°C, 30 s at 55°C, and 30 s at 72°C, for 35 cycles.

### Cell Culture

Mouse aortic SMCs were isolated from the abdominal aorta (from the diaphragm to the bifurcation) in five-week-old wild-type male mice, as previously described[15,23], and maintained in Dulbecco's Modified Eagle Medium (DMEM) with 10–20% fetal bovine serum (FBS). Cells were growth arrested at 70–80% confluence via incubation in serum-free medium for 48 hours before stimulating cells with rh-TRAIL (CD253; AbD Serotec). Different concentrations of EPA sodium salt (Sigma Aldrich) in 1% fatty acid-free bovine serum albumin (BSA; Calbiochem) in serum-free medium were applied to cells two hours before rh-TRAIL induction. Different concentrations of GW9508 (Cayman chemical) for activation of Gpr-120/Ffar-4, and either AS-601245 (Enzo Life Sciences) or SP600125 (Calbiochem), for inhibition of JNK activity, were added two hours before rh-TRAIL induction. *Gpr-120/Ffar-4* siRNA (Ambion silencer select) for knock-down was applied 24 hours before rh-TRAIL induction.

### Western Blotting

Cell lysates were prepared from growth-arrested vascular SMCs (VSMCs), which were stimulated with rh-TRAIL and then collected in radioimmunoprecipitation assay (RIPA) lysis buffer by scraping. Samples containing equal amounts of proteins were separated by sodium dodecyl sulfate polyacrylamide gel electrophoresis (SDS-PAGE) and transferred to a nitrocellulose membrane. The blot was incubated with a primary antibody (Phospho-SAPK/JNK, rabbit monoclonal, 1:1000, Cell Signaling; SAPK/JNK, rabbit monoclonal, 1:1000, Cell Signaling; Phospho-TAK-1, rabbit monoclonal, 1:1000, Cell Signaling; TAK-1, rabbit monoclonal, 1:1000, Cell Signaling) and subsequently with an appropriate secondary antibody. Signals were visualized with the enhanced chemiluminescence (ECL) system (Amersham-Pharmacia Co, U.K.). Images were captured using Photoshop (Adobe) and densitometry was performed using Image J software (NIH).

### Statistical Analysis

All data were statistically evaluated using Ekuseru-Toukei 2012 software (Social Survey Research Information Co., Ltd.) and quantitative values are expressed as mean ± standard deviation (SD). For mouse studies, a non-parametric analysis, Kruskal-Wallis test with Scheffe’s post-hoc analysis or Steel-Dwass post-hoc analysis, was performed. For cell culture experiments, which were performed independently three times, the Mann-Whitney *U* test was used for comparisons. For all statistical tests, P<0.05 was considered significant.

## Results

### EPA Attenuates the Development of AAAs in *Opg*-KO Mice

**Fig 1:**
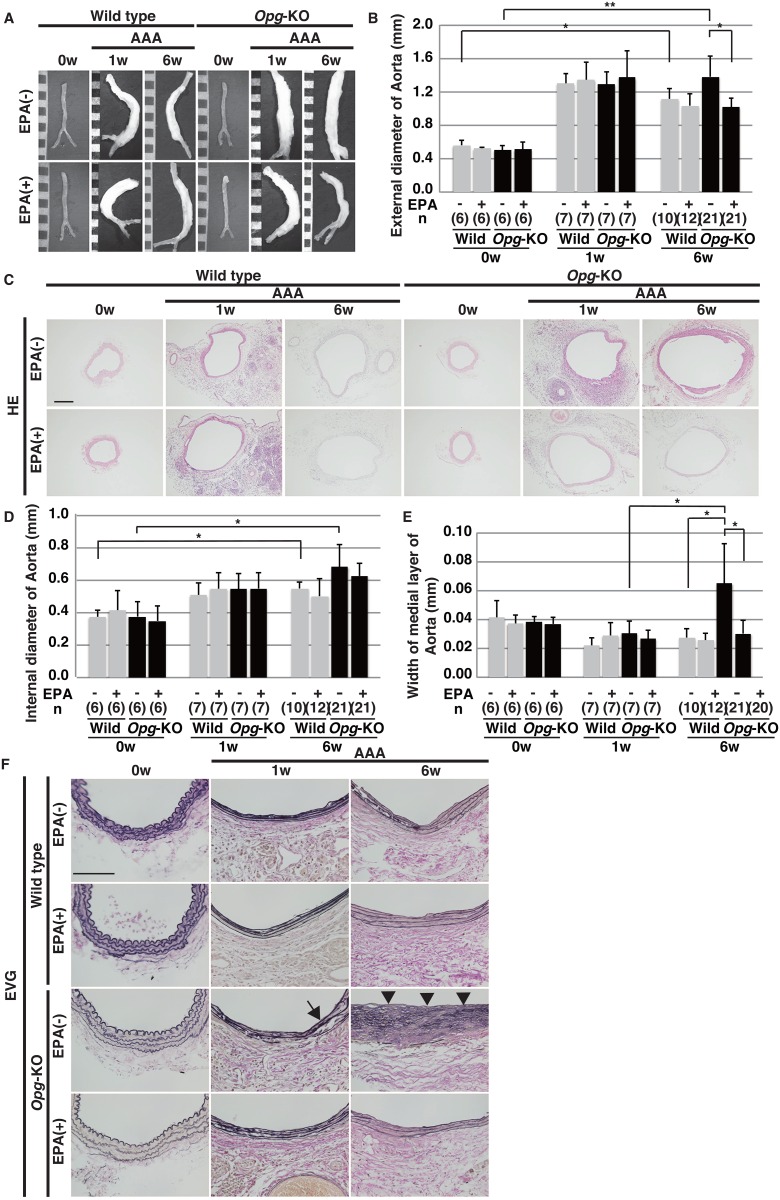
EPA attenuates progressive dilatation of the abdominal aorta in *Opg*-KO mice. **(A)** Representative aortas are shown at zero, one, and six weeks (w) after application of CaCl_2_ (AAA) in wild-type and *Opg*-KO mice, which were fed diets with (+) or without (-) EPA. Scale bars indicate 1 mm. **(B)** Measurements of the external aortic diameter at zero, one, and six weeks after AAA induction with the indicated diet conditions. **(C)** Representative H&E stained sections of aortas are shown. Scale bars indicate 200 μm. (**D, E)** Measurements of the internal aortic diameter **(D)** and the width of the medial layer **(E)** at zero, one, and six weeks after AAA induction. **(F)** Representative EVG-stained sections are shown. Collapse of elastic fibers (arrow) at one week and disruption of elastic lamella (arrowheads) at six weeks are observed in the medial layers of *Opg-*KO mice. Scale bars indicate 100 μm. Data are presented as mean ± SD. The sample number (n) is shown in parentheses. *p<0.05 or **p<0.01, compared at zero, one, and six weeks, with EPA (-) and (+) diets.

In order to examine whether EPA attenuates the development of AAA in *Opg-*KO mice, we first measured the size of the aorta in wild-type and *Opg*-KO mice, which were fed a diet with (+) or without (-) EPA starting two weeks before AAA induction[7], [15]. In wild-type mice, regardless of feeding with EPA or not, a significant increase in external aortic diameters was observed at six weeks after AAA induction (Fig 1A and 1B). No significant difference was found in external aortic diameters with or without EPA. In *Opg*-KO mice, the external aortic diameter was increased at one week. At six weeks, however, obvious enlargement of the external aortic diameter was observed in mice fed the EPA (-) diet, while this enlargement was significantly reduced in mice fed the EPA (+) diet (Fig 1A and 1B). No significant change in mouse body weight by EPA was observed (S1A Fig). Sham operations did not result in significant changes at any time point (S1B and S1C Fig).

Hematoxylin and eosin staining of aortic sections showed an increase of the internal diameter of the abdominal aorta and thinning of the medial layer at one week in wild-type and *Opg*-KO mice fed EPA (-) and (+) diets (Fig 1C–1E). Six weeks later, an increase of the internal diameter was maintained in both wild-type and *Opg*-KO mice. The width of the medial layer was markedly enlarged in *Opg*-KO mice fed the EPA (-) diet. This enlargement was significantly reduced with the EPA (+) diet (Fig 1C and 1E). Elastica van Gieson staining showed typical wavy lamellae of elastic fibers in the medial layers of wild-type and *Opg-*KO mice at day 0 (Fig 1F). In *Opg-*KO mice fed the EPA (-) diet, disruption of the medial elastic lamellae was observed at one week after AAA induction; at six weeks, medial elastic lamellae were completely disrupted. However, in *Opg*-KO mice fed the EPA (+) diet, elastic fibers were retained with flattened structures (Fig 1F). This suggests that EPA attenuates the adverse effects of the loss-of-function of *Opg* on the extracellular matrix of the aortic media.

#### EPA Reduces JNK Phosphorylation and Mmp-9 Expression in the Media of AAAs

**Fig 2:**
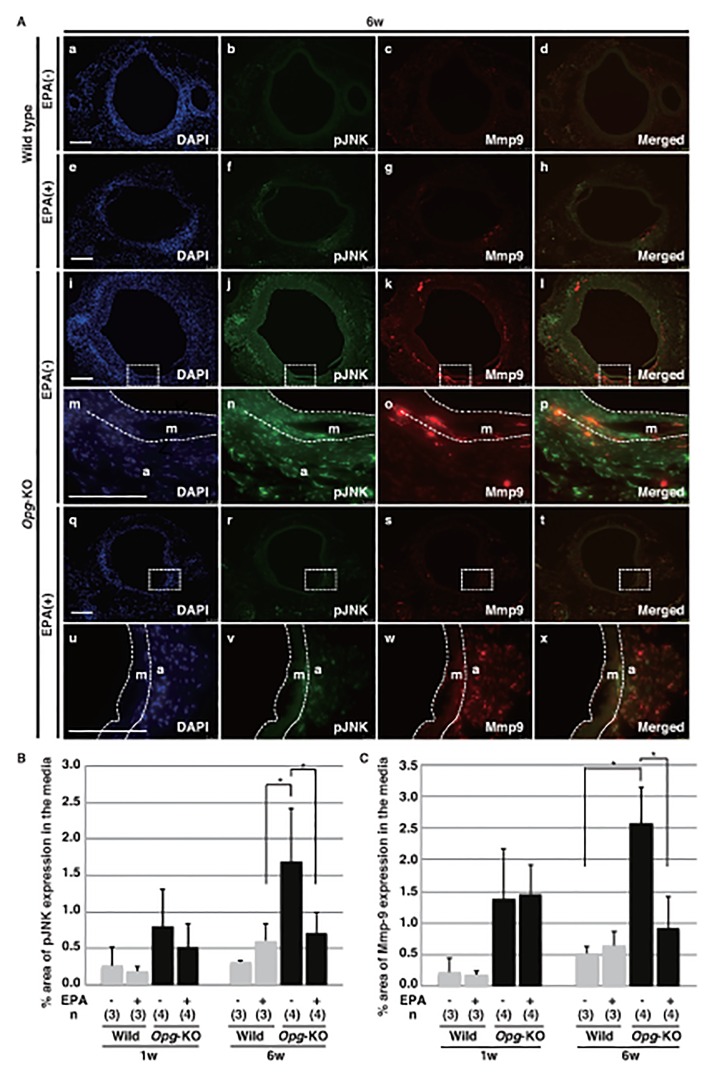
EPA attenuates the upregulation of Mmp-9 co-localized with pJNK in *Opg-*KO mice. **(A)** Representative double immunofluorescent staining images are shown for pJNK (green) and Mmp-9 (red) at six weeks (w) after AAA induction in the aortas of wild-type and *OPG*-KO mice fed EPA (+) and (-) diets. Rectangles with dotted lines in panels i-l and q-t are magnified in panels m-p and u-x, respectively. The border of the medial layer (m) with the intima or adventitia (a) is indicated by a white dotted line in panels m-p and u-x. Nuclei are stained with DAPI (blue). Scale bars represent 100 μm. **(B, C)** Percentages of stained areas for pJNK **(B)** and Mmp-9 **(C)** in the medial layers were calculated at one and six weeks for each diet condition. Data are presented as mean ± SD. The number of samples for analysis (n) is shown in parentheses. *p<0.05, as compared with EPA (-) diet conditions.

We next assessed JNK phosphorylation and Mmp-9 expression in the AAA model. Consistent with a previous report [15], focal expression of pJNK co-localized with Mmp-9 was observed in *Opg*-KO mice fed the EPA (-) diet at six weeks (Fig 2A). However, these findings were attenuated in the medial layer of *Opg*-KO mice fed the EPA (+) diet. Mmp-9 and pJNK double-positive areas were significantly increased in *Opg*-KO mice fed the EPA (-) diet compared to mice fed the EPA (+) diet (Fig 2B and 2C).

#### EPA Down-regulates Trail in the Media of AAAs

**Fig 3:**
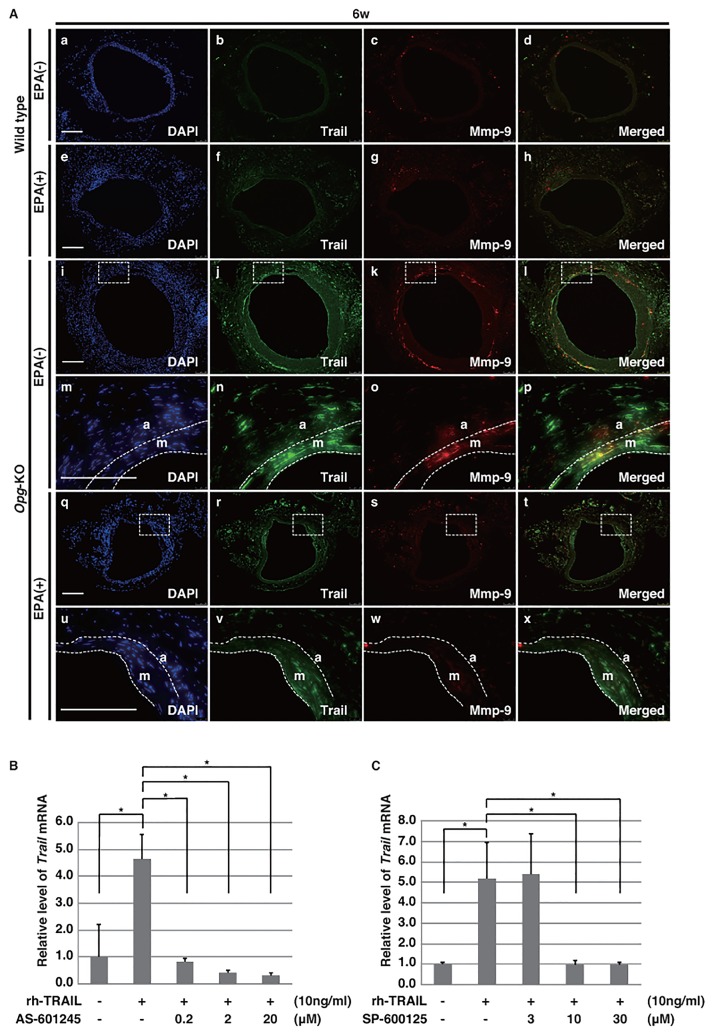
EPA prevents the up-regulation of Trail in *Opg-*KO mice via inhibition of JNK. **(A)** Representative double immunofluorescent staining images for Trail (green) and Mmp-9 (red) are shown at six weeks after AAA induction in the aortas of wild-type and *Opg*-KO mice fed an EPA (+) diet. Rectangles with dotted lines in panels i-l and q-t are magnified in panels m-p and u-x, respectively. The border of the medial layer (m) with the intima or adventitia (a) is indicated by a white dotted line in panels m-p and u-x. Nuclei are stained with DAPI (blue). Scale bars represent 100 μm. **(B-C)** In the SMC culture system, *Trail* mRNA expression induced by rh-TRAIL (10 ng/mL) was inhibited by JNK inhibitors AS-601245 (0.2–20 μM) **(B)** and SP-600125 (3–30 μM) **(C)**. Data are presented as mean ± SD; *p<0.05, as compared with controls or Trail alone. Experiments were independently repeated three times.

Trail is up-regulated in the media of AAAs in *Opg*-KO mice [15], and thus we tested whether Trail expression could be reduced with the EPA (+) diet. In *Opg*-KO mice fed the EPA (-) diet, Trail was markedly up-regulated in the outer zone of the medial layer, where Mmp-9 was mostly co-localized (Fig 3A). EPA suppressed the up-regulation of Trail and Mmp-9 in the media of *Opg*-KO mice (Fig 3A and S2 Fig).

We previously reported that Trail could induce its own expression, similar to TNF-α [24], and that its activation could be blocked by the decoy receptor Opg. Given that Trail and p-JNK are expressed in the medial layer, we hypothesized that Trail could induce its own expression through the JNK signaling pathway, and examined whether rh-TRAIL-induced *Trail* expression is inhibited by JNK inhibitors (AS-601245, SP-600125) in our SMC culture system. *Trail* expression was reduced in a dose-dependent manner (Fig 3B and 3C), suggesting that Trail induces its own expression via JNK activation in SMCs.

#### EPA Down-regulates *Mmp-9* Expression by Inhibiting the JNK Pathway in SMCs

**Fig 4:**
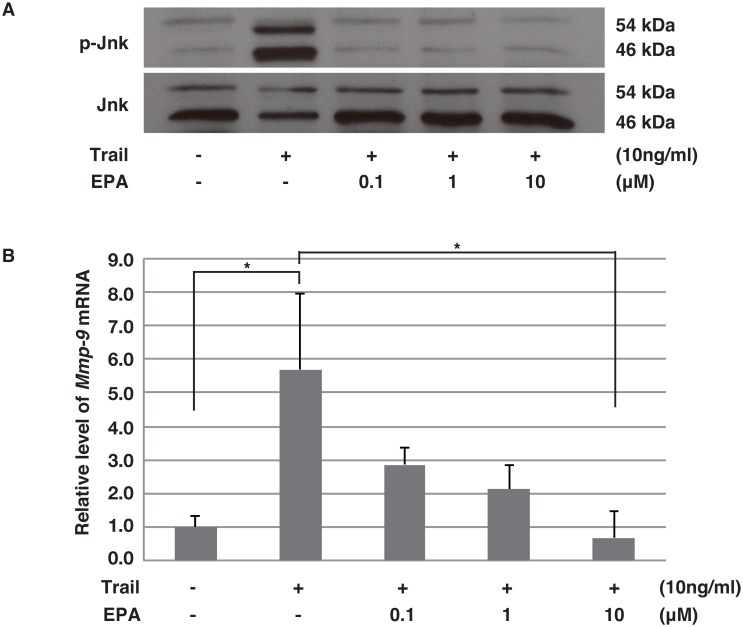
EPA reduces Trail-induced Mmp-9 expression via JNK. (**A**) Western blotting analysis for pJNK/JNK from SMCs, which were cultured with rh-TRAIL (10 ng/mL) for 30 minutes with or without pre-incubation with EPA (0.1–10 μM). (**B**) Relative expression levels of *Mmp-9* mRNA by qPCR from SMCs cultured with rh-TRAIL induction (10 ng/mL) for six hours with or without pre-incubation with EPA (0.1–10 μM). Data are presented as mean ± SD; *p<0.05, as compared with controls or Trail alone.

Up-regulation of *Mmp-9* expression by rh-TRAIL and TNF-α can occur through the JNK pathway[8], [15]. Here, we tested whether EPA could inhibit the increase in *Mmp-9* expression induced by Trail through JNK phosphorylation in SMCs. EPA reduced Trail-induced JNK phosphorylation (Fig 4A) and *Mmp-9* expression (Fig 4B).

#### EPA may Prevent AAAs through Gpr-120/Ffar-4 Receptors

**Fig 5:**
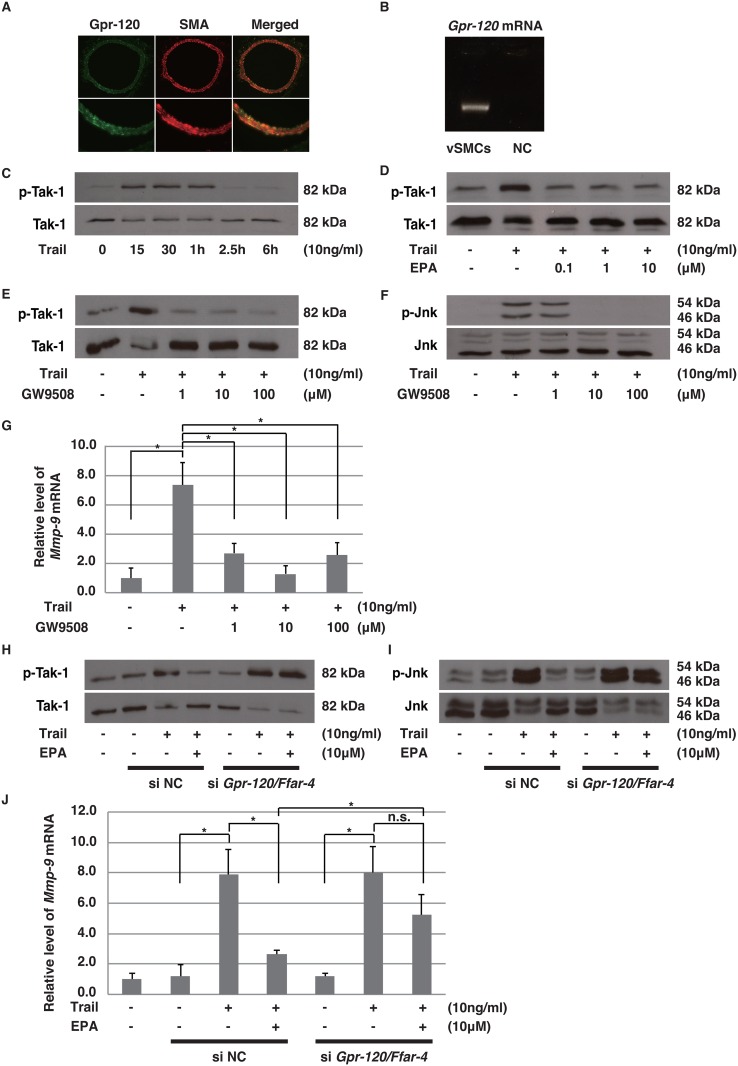
EPA reduces Mmp-9 expression by inactivating the Tak1-JNK pathway via activation of Gpr-120/Ffar-4. (A) Representative double immunofluorescent staining image shows Gpr-120/Ffar-4 (green) and α-SMA (red) in the aortas of wild-type mice. Lower panels are magnified from upper panels. **(B)** Amplified *Gpr-120/Ffar-4* mRNA samples derived from SMCs are shown. (**C-F**) Western blotting analysis for pTak-1/Tak-1 (**C-E**) and pJNK/JNK (**F**) from SMCs cultured for the indicated periods **(C)**, 15 minutes **(D and E)**, and 30 minutes **(F)** with rh-TRAIL (10 ng/mL). Phosphorylation of Tak-1 and JNK from SMCs, which were cultured with rh-TRAIL, was inhibited by EPA (**D**) and GW9508 (**E and F**). (**G**) Relative expression levels of *Mmp-9* mRNA in SMCs cultured for six hours with rh-TRAIL (10 ng/mL). Expression of *Mmp-9* in SMCs, induced by rh-TRAIL, were inhibited by GW9508. (**H-J**) Western blotting analysis for pTak-1/Tak-1 (**H**) and pJNK/JNK (**I**) from SMCs, which were treated with scrambled (NC) or *Gpr-120/Ffar-4* siRNA (10 nM) and then cultured with rh-TRAIL (10 ng/mL) for 15 minutes **(H)** and 30 minutes **(I)**. (**J**) Relative expression levels of *Mmp-9* mRNA from SMCs treated with scrambled (NC) or *Gpr-120/Ffar-4* siRNA (10 nM) and cultured for six hours with rh-TRAIL (10 ng/mL). Data are presented as mean ± SD; *p<0.05, as compared with controls or Trail alone.

To investigate the mechanism by which EPA reduces Trail-induced JNK phosphorylation and *Mmp-9* expression, we hypothesized that EPA might function as a ligand for Gpr-120/Ffar-4 and repress JNK signaling. We first examined whether Gpr-120/Ffar-4 is expressed in SMCs. Immunohistochemical analysis revealed that Gpr-120/Ffar-4 co-localizes with α-SMA in aortic tissue (Fig 5A). Moreover, *Gpr-120/Ffar-4* mRNA was detected in SMCs, as determined by RT-PCR analysis (Fig 5B). These findings confirm the expression of Gpr-120/Ffar-4 in SMCs.

EPA has been reported to exert its anti-inflammatory effects by inhibiting the activation of Tak-1, a kinase for JNK and NF-κB, through Gpr-120/Ffar-4[21]. We first confirmed that Trail activates Tak-1 (Fig 5C). We then tested whether EPA or GW9508, an agonist for Gpr-120/Ffar-4, could reduce the Trail-induced phosphorylation of Tak-1. Tak-1 phosphorylation was completely inhibited with EPA or GW9508 treatment (Fig 5D and 5E). In addition to Tak-1 inactivation, JNK phosphorylation and *Mmp-9* expression were inhibited by GW9508 in a dose-dependent manner (Fig 5F and 5G). Thus, stimulation of Gpr-120/Ffar-4 by EPA or GW9508 could inhibit the JNK pathway through inactivation of Tak-1.

To confirm whether Gpr-120/Ffar-4 is required for the effects of EPA, we knocked down *Gpr-120/Ffar-4* using siRNA (S3 Fig). Knockdown of *Gpr-120/Ffar-4* abrogated the inhibitory effects of EPA on Trail-induced phosphorylation of Tak-1 and JNK (Fig 5H and 5I) and Mmp-9 expression (Fig 5J). These data suggest that the activation of Gpr-120/Ffar-4, induced by EPA, may be crucial for inhibiting JNK phosphorylation and reducing *Mmp-9* expression.

## Discussion

In this study, we found that EPA prevents disruption of the media in the abdominal aorta of *Opg-*KO mice. We previously reported that CaCl_2_-induced inflammation was prolonged in *Opg*-KO mice, and that this resulted in complete destruction of the media[15]. We speculated that, in the absence of Opg, Trail-induced Trail expression forms a vicious cycle that strongly induces Mmp-9 expression. Here, we demonstrated that this vicious cycle is blocked by EPA. Upon investigation of the mechanism underlying this effect, we found that EPA reduces the phosphorylation of Tak-1 and JNK, and down-regulates *Mmp-9* expression via Gpr-120/Ffar-4 in cultured SMCs. Epidemiological studies have shown EPA to have inhibitory effects on cardiovascular events, especially in more severe cases[18]. We speculate that, in such cases, the function of OPG might be down-regulated or blocked, and that this insufficiency in OPG activity might worsen the vicious cycle of Trail-induced Trail expression. Our findings suggest that OPG, a protector of bone metabolism, also plays a protective role in vascular walls, and that EPA can rescue cells from the negative effects of OPG insufficiency.

Recent reports from two groups suggest that EPA plays a preventive role against AAA formation in different animal models. Wang et al. reported that EPA reduced TNF-α-induced MMP-9 expression in macrophages [19], and Yoshihara et al. found that EPA reduced the levels of inflammatory cytokines, such as IL-6 and MCP-1, in macrophages[20]. However, neither group focused on Trail, an inflammatory cytokine, nor did they address the role of SMCs in AAA formation. Our present study, for the first time, demonstrates a potential role for EPA in preventing AAAs and down-regulating Trail in SMCs.

Although EPA blocked severe AAA lesions in *Opg-*KO mice, it did not affect the formation of AAAs in wild-type mice (Fig 1B). Contrary to our results, Wang et al. showed a significant reduction of CaCl_2_-induced AAAs with EPA treatment. Because CaCl_2_-induced AAA formation is quite drastic, we decided to weaken the effects of CaCl_2_ by reducing the amount applied. Specifically, we restricted the contact area by using a small piece of cotton, and limited the treatment duration to 15 minutes. As a result, AAA lesions were weak in wild-type mice, to the extent that no differences were observed between EPA (-) and EPA (+) groups. Wang et al. also used two times higher dose of EPA (10% wt/wt) than that we used in this study. These different experimental conditions might explain the differences between their results and ours.

A mechanism underlying the effects of EPA has been previously proposed. First, activation of Gpr-120/Ffar-4 recruits β-arrestin-2 from the intercellular domain to the plasma membrane, and this is accompanied by the translocation of TAK-1/MAP3K7 binding protein 1 /MAP3K7IP1 (Tab-1), as a binding partner of β-arrestin-2, in macrophages and adipocytes[22]. Subsequently, Tak-1 and its downstream signaling pathway, including JNK and NF-κB, fails to be activated even in the presence of TNF-α or lipopolysaccharides (LPSs), given the lack of Tab-1 to activate Tak-1. Thus, EPA may inhibit the enlargement of AAAs through activation of Gpr-120/Ffar-4 by inactivating Tak-1 and the downstream JNK pathway in SMCs. This is consistent with a study by Yoshimura et al. that reported the prevention of AAA progression by inhibition of JNK[7].

Based on our results, we propose a model for the inhibitory effects of EPA on AAAs via SMCs (S4 Fig). EPA reduces Trail-induced *Mmp-9* expression by inhibiting the Tak-1-JNK pathway through Gpr-120/Ffar-4, thereby preventing the enhancement of AAAs. Our findings may contribute the discovery of therapeutics that suppress the dilatation of aortic aneurysms.

## Supporting Information

S1 Fig EPA does not influence body weight in both *Opg*-KO and wild type mice, and aortic diameter in sham operations. **(A)** Measurements of body weight at zero, one, and six weeks after AAA induction with the indicated diet conditions (n = 6–12). **(B)** Representative images of aortic samples at zero, one, and six weeks after saline application (sham operation) in wild-type and *Opg*-KO mice fed EPA (+) or (-) diets (n = 2–6). Scale bars indicate 1 mm. **(C)** Measurements of the external aortic diameter at zero, one, and six weeks after AAA induction with the indicated diet conditions.
(EPS)

S2 Fig EPA prevents the up-regulation of Trail in *Opg-*KO mice. The percentage of stained areas for Trail in the medial layers was calculated at six weeks for each diet condition. Data are presented as mean ± SD. The number of samples for analysis (n) is shown in parentheses. *p<0.05, as compared with EPA (-) diet conditions.
(EPS)

S3 Fig siRNA-mediated knock-down of *Gpr-120/Ffar-4* expression. Relative expression levels of *Gpr-120/Ffar-4* mRNA by qPCR from SMCs cultured with rh-TRAIL (10 mg/mL) for six hours with or without pre-incubation with EPA (10 μM). *Gpr-120/Ffar-4* siRNA was applied 24 hours before pre-incubation with EPA. Data are presented as mean ± SD; *p<0.05, as compared with control.
(EPS)

S4 Fig Schematic of mechanism by which EPA prevents the development of AAAs. In *Opg-*KO mice, inflammation triggered by CaCl_2_ induces the up-regulation of inflammatory cytokines, such as Trail, in SMCs of the aorta. Trail activates the Tak-1-JNK pathway through the Trail receptor (Trail-R), inducing the expression of genes encoding proteolytic enzymes for the extracellular matrix, such as *Mmp-9* and *Trail* itself. The enhanced activity of proteolytic enzymes normally allows for remodeling of the aortic walls, leading to aneurysmal dilatation in wild-type mice. On the other hand, with the loss of *Opg* function, continuous expression of Trail and excessive production of Mmp-9 occur in SMCs. This condition could be a major cause of the disruption of medial elastic fibers. EPA binds to and activates Gpr-120/Ffar-4 as a ligand and inhibits Tak-1, resulting in the inactivation of JNK and reduced expression of *Mmp-9*.
(EPS)
